# The influence of depressive symptoms on the effectiveness of a short-term group form of Schema Cognitive Behavioural Therapy for personality disorders: a naturalistic study

**DOI:** 10.1186/s12888-020-02676-z

**Published:** 2020-06-01

**Authors:** David Koppers, Henricus Van, Jaap Peen, Jet Alberts, Jack Dekker

**Affiliations:** 1 Department of Research and Quality of Care, ARKIN Mental Health Institute, Klaprozenweg 111, 1033 NN Amsterdam, The Netherlands; 2 ARKIN Mental Health Institute, NPI, Centre for Personality Disorders, Domselaerstraat 128, 1093 MB Amsterdam, The Netherlands; 3 Faculty of Behavioural and Movement Sciences, Clinical Psychology Section, VU-University Amsterdam, Van der Boechorstraat 7, 1081 BT Amsterdam, The Netherlands

**Keywords:** Personality disorders, comorbidity, Schematherapy, Group psychotherapy, treatment effectiveness

## Abstract

**Background:**

This naturalistic study examined the outcomes of Short-Term Schema Cognitive Behavioural Therapy in groups with personality disorders, and with high and low severity of depressive symptoms.

**Methods:**

Assessments were made at baseline, at mid-treatment (week 10), at treatment termination (week 20) and at three-month follow-up (week 32) of 225 patients with personality disorders and high severity of depressive symptoms (PD-Hi) and patients with low severity of depressive symptoms (PD-Lo). The assessments focused on symptom (Symptom Checklist-90) and schema severity (Young Schema Questionnaire) and coping styles (Utrecht Coping List). We also measured the rate of symptom remission. The data obtained were subjected to multilevel analysis.

**Results:**

Psychiatric symptoms and maladaptive schemas improved in both patient groups. Effect sizes were moderate, and even small for the coping styles. Symptom remission was achieved in the minority of the total sample. Remission in psychiatric symptomatology was seen in more PD-Lo patients at treatment termination. However, the difference in levels of remission between the two patient groups was no longer apparent at follow-up.

**Conclusion:**

A short-term form of schema therapy in groups proved to be an effective approach for a broad group of patients with personality disorders*.* However*,* the majority of patients did not achieve symptom remission.

**Trial registration:**

Not applicable.

## Background

The efficacy of individual psychotherapy for patients with personality disorders (PD) has been well demonstrated in several meta-analyses [1–4].

Nevertheless, group psychotherapy is frequently advocated as an alternative to individual approaches because the symptoms of PD become apparent at an interpersonal level and it is therefore conceivable that they could be addressed more effectively during group interactions [5].

A few studies [5–8] of short forms of group psychotherapy have indeed shown that patients could benefit. In addition, a group approach could be a cost-effective way to treat patients [9] and help to cut the long waiting lists for many PD services.

The schema therapy approach has been extended to personality disorders other than borderline personality disorders only [10] and to group therapies. We have found two naturalistic studies of effectiveness for personality disorders after a short form of group psychotherapy (20 sessions) in an outpatient setting: van Vreeswijk et al. [11] and Renner et al. [7] found a moderate (SCL-90-GSI; *d* = 0.66) and large effect size (SCL-90-GSI; *d* = 0.81) respectively. However, most outcome studies of the treatment of personality disorders have failed to look at how comorbidity affects outcome, even though, in daily practice, many patients suffer from comorbid conditions, generally depressive disorders [12]. Nevertheless, we have found only two studies [13, 14] that examine the impact of depressive symptoms on the treatment of personality disorders. Hellerstein et al. [13] found that comorbid dysthymic disorders impaired remission from personality disorders in long-term individual treatment. Renner et al. [14] found that patients with comorbid depression had more severe psychiatric and personality pathology at baseline and poorer treatment outcome after long-term individual schema therapy. However, this was not due to comorbid depression but to the significantly higher general psychiatric symptomatology at baseline in patients with a personality disorder and comorbid depression. This finding suggests that severe baseline psychiatric pathology could be a strong predictor of treatment outcome.

As far as we know, the effect of comorbid depression on the outcome of short-term group therapy for PD has never been examined.

We therefore studied the effectiveness of short-term Schema Cognitive Behavioural Therapy in groups (SCBT-g) [15] in an open cohort of patients with a personality disorder with low severity of depressive symptoms (PD-Lo) and patients with a personality disorder with high severity of depressive symptoms (PD-Hi). Our aim was to determine the role of depressive symptoms on psychiatric symptom severity at baseline, during treatment at treatment outcome and three-month follow-up. Patients had one or more PD diagnoses and were all referred to a specialised service for the treatment of PD.

Our research questions were:

1. Are there pre-treatment differences between the sociodemographic and clinical characteristics of PD patients with high or low severity of depressive symptoms?
2. Could we identify relevant differences in the effects of therapy in personality disorder patients with high or low severity of depressive symptoms at treatment termination and at follow-up?

## Methods

### Study design

The current study used an open pre-post intervention design [16]. Patients were recruited from January 2012 through to December 2017 at the NPI Centre for Personality Disorders, a specialised service for PD treatment that is part of the Arkin mental health institute in Amsterdam. The study was granted an exemption from the provisions of the Medical Research Involving Human Subjects Act (WMO) by the Medical Ethics Review Committee of VU-University Medical Center in Amsterdam and approved by the ethics board of the mental health institute ARKIN in Amsterdam. All patients in the study gave informed consent.

#### Participants

Patients were referred to the NPI Centre for Personality Disorders by their general practitioner. The NPI has a treatment programme consisting of three treatment pathways which differ in terms of treatment intensity and duration. Each pathway consisted of a number of treatment modalities, mainly from either a psychodynamic or a schema-focused perspective. After a clinical intake and a process of shared decision-making, patients are referred to one of the treatment modalities of these treatment pathways.

The Schema Cognitive Behavioural Therapy in groups (SCBT-g) is part of the short-term treatment pathway focusing on personality change (< 1 year treatment). Our study targets those patients who have followed the SCBT-g treatment modality only. The inclusion criteria for SCBT-g were: age 18 to 65 years and fulfilment of the DSM-IV criteria for at least one PD. The diagnosis was made in clinical interviews. The exclusion criteria were: severe suicidality, antisocial personality disorder, severe somatic problems/illness, acute and disruptive psychosocial problems such as homelessness, no income or high debts and inability to participate in a group due to communication problems (stuttering, deafness or language barrier).

During the study period (January 2012 to December 2017), approximately 1100 patients were referred to the short-term treatment pathway oriented towards personality change. Of these patients, 225 (20.5%) were selected for the SCBT-g modality on the basis of the inclusion criteria listed above and after a shared decision-making process that could also involve practical considerations such as the availability of groups or the times at which the patient was available to attend therapy etc.

### Intervention

The SCBT-g is a highly structured group therapy format based on the protocol by Broersen and van Vreeswijk [15]. It consists of twenty weekly sessions of group therapy with 8 or 9 patients. Every session lasts 2 h, including a short break. The programme comprised two phases: the conceptualisation phase and the schema-change phase. In the conceptualisation phase, the patients identified their three main schemas by discussing the results from the Young Schema Questionnaire, through psycho-education about the schema model and by discussing the origins of the patients’ schemas. The schema-change phase consists of interventions focused on challenging and changing the maladaptive schemas and schema behaviour into more adaptive schema behaviour patterns with cognitive modification techniques, behaviour experiments and experiential interventions. Before the start of the group therapy, the patients were invited to attend two individual introduction sessions at which the SCBT-g was explained and a final eligibility check took place. Evaluation sessions were individual and took place at mid-treatment (week 10), treatment termination (week 20) and 3 months after the end of therapy (week 32).

During the study period, 26 therapists worked in pairs with a total of 31 parallel groups.

Each group had one pair of therapists, with at least one therapist being a general mental health psychologist. Thirteen therapists were general mental health psychologists, one was a clinical psychologist, two were psychiatrists, two were psychotherapists, two were resident psychiatrists, one was a resident clinical psychologist and five were social psychiatric nurses.

All therapists completed a 56-h course in schema therapy and at least 50 h of group supervision for schema therapy chaired by a schema therapist registered as a supervisor with the Dutch Association of Schema Therapy. In addition, all therapists attended a weekly peer supervision session lasting 1 h.

### Measurements

#### Baseline assessments

##### Personality disorder

Before the intervention in question, patients were assessed in a standard intake procedure (i.e. clinical interview) conducted by government-registered psychologists or psychiatrists. The intake procedure comprised a consistent interview schedule consisting of two parts, the first to make a general evaluation of the patient’s psychopathology, the second to establish a biography for the patient. Insurance requirements meant that only patients with a confirmed DSM-IV [17] personality disorder diagnosis could be treated in the NPI Centre for Personality Disorders.

##### Comorbid depressive symptoms

The severity of comorbid depressive symptoms was measured with the Symptom Check List depression scale (SCL-90-R). On the basis of the Dutch norms for an outpatient psychiatric population [18] for the Symptom Checklist-90-R, a cut-off of 48 points (in other words, an above-average score) was used, above which patients were considered to have a high severity of depressive symptoms. As a consequence, we have labelled patients with a score below the cut-off score of 48 points (in other words, an average score to very low score) ‘the patient group with a personality disorder and a low severity of depressive symptoms’ (the PD-Lo group) and patients with a score of 48 points or above ‘the patient group with a personality disorder and a high severity of depressive symptoms’ (the PD-Hi group).

##### Measurement instruments

All measurement instruments for outcome were completed by the patients at baseline, after 10 weeks, at treatment termination (20 weeks) and at three-month follow-up (32 weeks). The data were collected and classified by trained research assistants (master-level graduate students in clinical psychology).

The following measurement instruments were used:

##### The symptom checklist 90-revised

The Symptom Checklist 90-Revised (SCL-90-R) [19] Dutch translation [18], a self-report instrument, consists of 90 items covering different symptom scales rated from ‘1, not at all’ to ‘5, could not be worse’. The scales are: *anxiety*, *phobic anxiety*, *depression*, *somatisation*, *insufficiency*, *interpersonal sensitivity*, *hostility*, *sleep problems*, and a *Global Severity Index* (GSI) scale. This last scale is the mean for all items. The instrument is well validated and internal consistency is high (Cronbach α = .82–.97). Test-retest reliability is good [20]. The internal consistency in this study is high (SCL-90 Cronbach α = 0.98; subscales SCL-90 = 0.80–0.94).

##### The Young Schema Questionnaire

The Young Schema Questionnaire (YSQ) [21] Dutch version [22] is a 205-item self-report questionnaire that is scored on a six-point Likert scale. It is used to measure 16 maladaptive schemas (core beliefs) as defined by Young et al. [23]. These sixteen schemas are grouped in five schema domains. *Schema domain 1* = *disconnection and rejection* (schemas: abandonment/instability, mistrust/abuse, emotional deprivation, social isolation and social undesirability), *Schema domain 2 = impaired autonomy* (dependency/incompetence, undeveloped self/enmeshment, defectiveness/shame, and failure to achieve), *schema domain 3 = impaired limits* (entitlement and insufficient self-control/discipline), *schema domain 4 = other directedness* (subjugation and self-sacrifice), *schema domain 5 = over-vigilance and inhibition* (emotional inhibition, unrelenting standards and vulnerability to harm/illness). Research has shown that, in the Dutch version of the YSQ, internal consistency is adequate to high in all schema scales (Cronbach α = 0.73–0.93) [24]. The internal consistency of the YSQ in this study is large (Cronbach α = 0.97).

##### The Utrecht coping list

The Utrecht Coping List (UCL) [25] is a self-report questionnaire for measuring cognitive and behavioural coping patterns in order to determine which characteristic coping style is used when confronting problems or complex situations. The UCL covers 47 items. The following seven scales were extracted by factor analysis from 44 scaled items: *active coping* (7 items), *palliative reaction pattern* (8 items), *avoidance* (8 items), *seeking social support* (6 items), *passive reaction pattern* (7 items), *expression of emotions* (3 items) and r*eassuring thought* (5 items). Each of the items is rated on a four-point scale from ‘Doesn’t apply not to me’; ‘Applies seldom to me’, ‘Applies often to me’ to ‘Applies to me’. The UCL has good psychometric properties. The internal consistency of the majority of the seven subscales is moderate (Cronbach’s α = 0.71–0.78). However, for the subscales *expression of emotions* and *reassuring thoughts*, internal consistency is low (Cronbach’s *α* = 0.55 and 0.60, respectively) [25]. For this study, internal consistency was moderate for four of the seven scales (Cronbach’s *α* = 0.70–0.86). For the scales *passive reaction pattern* (*α* = 0.68), *expression of emotions* (*α* = 0.64) and *reassuring thoughts* (α = 0.66), internal consistency was low. We present only the results of the following scales, for which the internal consistency alpha (Cronbach’s α > 0.70) is moderate or higher: *active coping*, *palliative reaction pattern*, *avoidance* and *seeking social support*. Reliability (*r* = 0.45–0.85) is moderate to reasonably good [25].

We measured outcomes in three areas: general symptom severity (General Severity Index scale (GSI) of the SCL-90-R); severity of maladaptive schemas (Young Schema Questionnaire) and coping styles (Utrecht Coping List for measuring coping mechanisms). Secondly, we determined treatment success with the two-step approach of Jacobson and Truax [26] based on pre- to post- and follow-up treatment changes on the SCL-90 Global Severity Index (GSI).

### Statistical analysis

Chi-square tests (categorical variables) and ANOVA (continuous variables) were used to compare the baseline characteristics of patients with and without comorbid depressive symptoms.

Within-group effect sizes (Cohen’s *d*) [27] were calculated at week 20 and at three-month follow-up (32 weeks).

Linear mixed-model analyses were used to analyse the repeated continuous outcomes. First, we evaluated the treatment effect for the total patient group, the PD-Lo group and the PD-Hi group. Subsequently, we examined the difference between the PD-Lo group and the PD-Hi group. These analyses were conducted using a two-level structure (patient, and repeated measurement occasion). As we used mixed-model analyses to evaluate outcome measures, no imputation of missing data was needed [28]. Time was treated as a categorical variable to assess the treatment effects at the end of treatment and at follow-up for the PD-Lo group by contrast with the PD-Hi group. In order to check for possible confounding, we added the following variables with a baseline difference between the two patient groups (*p* < 0.10; see Table 1) to all analyses: cultural background (Dutch, north-western countries or non-western countries), job status, prior treatment for current treatment and medication use (see Table 1). We also added gender and age as covariates because depression is more common among females and prevalence varies by age [29]. Demographics are stated as numbers and rates. With regard to cultural background, we assigned patients from the Netherlands to the category Dutch, patients from Northwest Europe, the United States, Australia and Canada to the category north-western countries and patients from other countries to the category non-western countries. With regard to job status, we allocated patients with employment problems due to sickness to the category sickness benefits/social security benefits.

**Table 1:** Baseline characteristics for the total sample, the PD-Lo patient group and PD-Hi patient group

Variable		Total sample (*n* = 225)	PD-Lo (*n* = 131)	PD-Hi (*n* = 94)	Test statistic (df)	*p*
**Demographics**						
Age n (mean)		225 (39.4)	131 (39.6)	94 (39.0)	F (1)= 0.229	.63
Gender n (%)	male	82 (36)	51 (39)	31 (33)	χ2(1) = .837	.36
Cultural background n (%)	Dutch	156 (69)	96 (73)	60 (64)	χ2(2) = 5.279	.07
	North-Western countries	19 (8)	15 (12)	8 (9)		
	Non-Western	46 (20)	20 (15)	26 (28)		
Marital status n (%)	single	157 (70)	91 (70)	66 (70)	χ2(2) = .259	.88
	married/living together	46 (20)	28 (21)	18 (19)		
	divorced	22 (10)	12 (9)	10 (11)		
Living situation n (%)	Living alone	152 (68)	88 (67)	64 (68)	χ2(4) = 2.231	.69
	Living alone with children	21 (9)	12 (9)	9 (10)		
	Living with parent/guardian	7 (3)	4 (3)	3 (3)		
	Living together	32 (14)	17 (13)	15 (16)		
	Living together with children	13 (6)	10 (8)	3 (3)		
Job status n (%)	Job	100 (44)	67 (51)	33 (35)	χ2(4) = 8.441	.08
	Student	25 (11)	12 (9)	13 (14)		
	Sickness benefits/Social security benefits	44 (20)	24 (18)	20 (21)		
	unemployed	48 (21)	22 (17)	26 (28)		
	Other	7 (4)	6 (5)	2 (2)		
Educational level n (%)	Low	106 (48)	64 (51)	42 (45)	χ2(2) = 0.718	.70
	Intermediate	72 (33)	40 (32)	32 (34)		
	High	41 (19)	22 (18)	19 (20)		
**Clinical characteristics**						
Symptom severity, SCL-GSI Mean (SD)		210.72 (58.05)	176.60 (36.56)	258.26 (48.20)	F [1] = 208.73	.00**
Schema severity, YSQ total Mean (SD)		2.95 (0.70)	2.64 (0.60)	3.39 (0.60)	F (1) = 86.168	.00**
Number personality disorders n (%)						
	one personality disorder	140 (62)	89 (68)	51 (54)	χ2(3) = 7.052	.13
	two personality disorders	33 (15)	18 (14)	15 (16)		
	three personality disorders	51 (23)	24 (18)	27 (29)		
	four personality disorders	12 (5)	4 (3)	8 (9)		
Specific personality disorder n (%)						
	borderline personality disorder	49 (22)	28 (21)	21 (22)	χ2(3) = 5.462	.70
	avoidant personality disorder	33 (15)	22 (17)	11 (12)		
	personality disorder not otherwise specified	126 (56)	71 (54)	55 (58)		
	other personality disorder	17 (7)	10 (8)	7 (8)		
Medication n(%)		109 (48)	55 (42)	54 (57)	χ2(1) = 5.239	.02*
Type of medication n(%)	antidepressants	75 (53)	37 (56)	38 (51)	χ2(3) = .561	.91
	antipsychotics	22 (16)	9 (14)	13 (17)		
	benzodiazepines	27 (19)	12 (18)	15 (20)		
	other mental	17 (12)	8 (12)	9 (12)		
Medication somatics n(%)		6 (5)				
Prior treatment for current treatment n(%)		202 (91)	113 (87)	89 (96)	χ2(1) = 4.895	.03*
Drop-out history n(%)		59 (28)	30 (24))	29 (33)	χ2(1) = 2.170	.14

*PD-Lo* Personality Disorder with low severity of depressive symptoms; *PD-Hi* Personality Disorder with high severity of depressive symptoms; North-Western countries (North-Western Europe, North-America, Australia) * *p* < .05, **p < .01

Remission rates for the SCL-GSI at end of treatment and follow-up were calculated on the basis of the two-step approach of Jacobson and Truax [26]. We broke down treatment success into two categories: Reliable Change Index and Remission. The Reliable Change Index describes significant improvement between two measurement occasions. Remission is a combination of a Reliable Change Index and a Clinically Significant Change (CSC). A CSC describes those patients who exceed a cut-off score based on Lambert, Hansen and Bauer [30]. Scores below these cut-off scores were classified as non-clinically significant. In the two-step approach of Jacobson and Truax [26], the Reliable Change Index is calculated first. The second step consists of determining whether patients who achieved reliable change also exceeded the cut-off score (Clinical Significant Change). The cut-off scores used in this study were a SCL-GSI score of 147.66 for the population as a whole, 141.90 for men and 153.73 for women. Patients who achieved reliable change and had a SCL-GSI score below the cut-off score were considered to have achieved remission.

## Results

### Flowchart

**Fig. 1:**
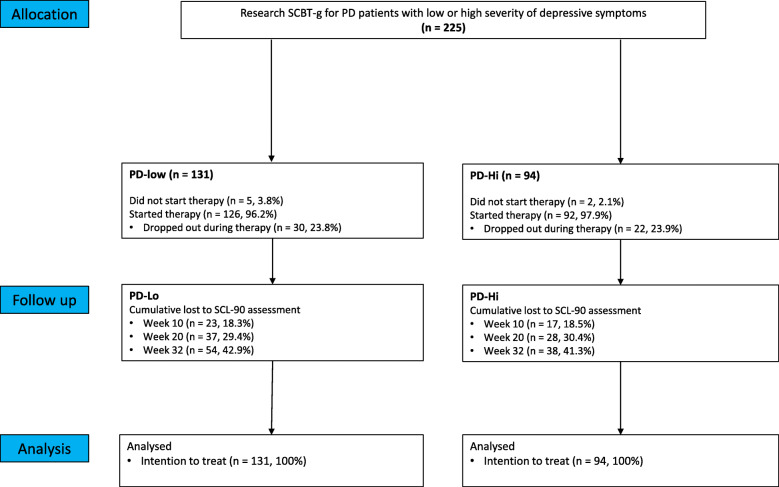
Flowchart

Figure 1 shows the flow for participants. All 225 patients met the inclusion criteria of the SCBT-g and were invited for the baseline assessment. Of the total sample, 94 patients (41.8%) had PD-Hi and 131 (58.2%) had a PD-Lo. Five patients (3.8%) in the PD-Lo group and two (2.1%) in the PD-Hi group refused the treatment intervention.

A total of 52 patients dropped out during treatment. There were no baseline differences between the patients who dropped out and the patients who completed the treatment.

Thirty (23.8%) patients in the PD-Lo sample and 22 in the PD-Hi sample (23.9%) dropped out. This difference was not significant (χ2(1) = 0.000, *p* = 0.99).

Thirty-seven (71%) of the patients dropped out during the first ten treatment sessions and 15 (29%) during the last ten sessions. There was no difference in time of drop-out between the two patient groups (χ2(1) = 0.583, *p* = 0.45). The main reason for drop-out was a loss of motivation (62%).

### Baseline characteristics

Table 1 shows the baseline sociodemographic and clinical characteristics. There were no differences between the sociodemographic characteristics of the PD-Lo and PD-Hi patient groups. The PD-Hi patients used more medication and had previously received mental health treatment more frequently than the PD-Lo patient sample.

The majority of the research sample had one personality disorder (DSM IV) [17]. More than 50% had an unspecified personality disorder. Almost 25% of the research sample was diagnosed with a borderline personality disorder. There were no differences between the two patient groups in terms of number and category of personality disorders.

The PD-Hi group had higher baseline scores for all SCL-90 scales and maladaptive schemas other than *depression*. The coping style scores indicated that PD-Hi patients made more use of the *avoidance* coping style and less of *active coping* and *seeking social support*. There was no difference in use between the two patient groups with regard to UCL palliative reaction pattern (F [1] = 0.843, *p* = 0.35).

### Effectiveness and impact of comorbidity on symptom distress, schema severity and coping styles

For the total sample, the mean scores on symptom severity (SCL-GSI) were 178.84 (58.99) (mean (SD)) at treatment termination and 180.47 (57.50) (mean (SD)) at three-month follow-up. The effect sizes were moderate at treatment termination (*d* = 0.50) and small at follow-up (*d* = 0.45).

According to the YSQ total, the mean scores for schema severity were 2.49 (0.75) (mean score (SD)) at treatment termination and 2.53 (0.86) (mean score (SD)) at three-month follow-up. The effect size was moderate (*d* = 0.56) at treatment termination and small at follow-up (*d* = 0.49).

Table 2 shows the mean and SD scores of the treatment for the PD-Lo group and PD-Hi group at baseline, mid-treatment (10 weeks), at end of treatment (20 weeks) and three-month follow-up (32 weeks).

**Table 2:** Change of symptoms, maladaptive schemas and coping styles at baseline, mid-treatment (week 10), treatment termination (week 20) and three-month follow-up (week 32)

	PD-Lo				PD-Hi			
	baseline	week 10	week 20	week 32	baseline	week 10	week 20	week 32
	(*n* = 131)	(*n* = 103)	(*n* = 89)	(*n* = 72)	(*n* = 94)	(*n* = 75)	(*n* = 64)	(*n* = 54)
**Outcome measure**	***M (SD)***	***M (SD)***	***M (SD)***	***M (SD)***	***M (SD)***	***M (SD)***	***M (SD)***	***M (SD)***
SCL scales								
*anxiety*	18.95 (5.55)	19.31 (7.63)	17.17 (6.78)	17.90 (6.93)	28.30 (7.86)	24.76 (8.27)	23.42 (8.91)	22.72 (8.16)
*phobic anxiety*	10.50 (3.56)	10.78 (4.19)	10.03 (4.29)	10.04 (3.94)	15.90 (6.40)	13.90 (6.91)	12.76 (5.66)	13.30 (6.83)
*depression*	35.17 (7.88)	36.32 (11.09)	31.09 (10.97)	33.94 (10.94)	56.95 (7.12)	47.31 (12.91)	42.61 (14.31)	42.70 (13.02)
*somatisation*	21.19 (6.94)	21.30 (7.67)	19,53 (6.60)	20.18 (7.29)	30.63 (9.75)	26.19 (8.61)	24.90 (9.72)	25.28 (8.77)
*insufficiency*	20.47 (5.81)	20.07 (6.59)	18,16 (6.42)	18.88 (7.05)	28.89 (6.55)	23.72 (7.76)	24.29 (7.95)	22.82 (7.76)
*interpersonal sensitivity*	36.34 (9.31)	37.25 (11.00)	31.55 (10.20)	32.71 (11.20)	52.76 (12.89)	44.35 (13.80)	42.23 (13.05)	41.38 (15.57)
*hostility*	10.27 (3.70)	9.95 (3.42)	8.81 (3.14)	8.67 (2.71)	13.80 (5.57)	11.72 (5.14)	11.45 (5.12)	10.88 (4.35)
*sleep problems*	7.16 (3.10)	6.97 (3.25)	6.29 (3.22)	6.99 (3.36)	9.18 (3.49)	8.24 (3.48)	7.49 (3.29)	7.76 (3.77)
*other*	16.71 (4.58)	16.68 (5.03)	14.61 (4.77)	15.00 (4.78)	22.71 (5.43)	19.82 (5.70)	18.76 (6.22)	17.38 (5.56)
*General Symptom Inventory*	176.60 (36.56)	178.84 (47.44)	157.11 (43.23)	163.59 (49.50)	258.26 (48.20)	220.15 (57.77)	209.54 (61.70)	204.44 (59.98)
YSQ domains								
*schema severity*	2.64 (0.60)	2.55 (0.64)	2.24 (0.68)	2.27 (0.75)	3.39 (0.60)	3.13 (0.69)	2.84 (0.70)	2.86 (0.87)
*disconnection-rejection*	2.74 (0.73)	2.66 (0.77)	2.31 (0.76)	2.29 (0.89)	3.60 (0.74)	3.34 (0.82)	3.03 (0.85)	3.07 (1.01)
*impaired autonomy*	2.34 (0.69)	2.28 (0.69)	1.99 (0.71)	1.99 (0.77)	3.12 (0.73)	2.85 (0.77)	2.53 (0.74)	2.58 (0.92)
*impaired limits*	2.72 (0.67)	2.57 (0.73)	2.27 (0.71)	2.25 (0.73)	3.22 (0.80)	3.00 (0.83)	2.81 (0.81)	2.74 (0.90)
*other directedness*	3.01 (0.77)	2.94 (0.78)	2.57 (0.84)	2.60 (0.90)	3.63 (0.76)	3.32 (0.79)	3.07 (0.77)	3.04 (0.95)
*overvigilance and inhibition*	2.57 (0.64)	2.46 (0.69)	2.19 (0.71)	2.20 (0.72)	3.33 (0.66)	3.04 (0.82)	2.79 (0.80)	2.74 (0.95)
UCL scales								
*active coping*	16.77 (4.16)	17.10 (4.29)	17.36 (3.83)	17.61 (3.75)	15.11 (4.47)	15.66 (4.39)	16.74 (4.33)	17.69 (4.15)
*avoidance*	17.65 (3.45)	18.03 (4.25)	17.11 (3.30)	17.60 (3.65)	19.46 (4.07)	18.35 (3.59)	18.16 (3.41)	18.65 (3.88)
*palliative reaction pattern*	18.50 (4.02)	19.04 (4.36)	18.15 (3.40)	17.70 (3.83)	19.04 (4.69)	19.51 (3.59)	18.55 (3.75)	19.20 (4.04)
*seeking social support*	13.17 (3.88)	14.16 (4.17)	14.17 (3.67)	13.86 (3.88)	12.01 (4.49)	12.87 (3.73)	13.39 (3.61)	13.59 (4.14)

*PD-Lo* Personality Disorder with low severity of depressive symptoms; *PD-Hi* Personality Disorder with high severity of depressive symptoms; *SCL* Symptom Checklist; *YSQ* Young Schema Questionnaire; *UCL* Utrecht Coping List; *M* Mean; *SD* Standard Deviation

The pre-post effect sizes on the SCL scales were small (*d* = 0.15–0.49) and small to large (*d* = 0.29–1.25) for the PD-Lo and the PD-Hi groups respectively.

The improvements in schema severity on the the YSQ domains were significant, with moderate effect sizes *d* = 0.44–0.56 and 0.40–0.72 for the PD-Lo and the PD-Hi groups respectively.

With regard to the coping styles, the pre-post effect sizes were 0.06–0.29 and 0.06–0.35 for the PD-Lo and PD-Hi groups respectively. The effects on the UCL scale *avoidance and palliative reaction pattern* were not significant in either patient group.

At follow-up, the effect sizes on the SCL scales were small (*d* = 0.04–0.43) and small to large (*d* = 0.18–1.28) for the PD-Lo and PD-Hi patient groups respectively. The effects were not significant in the PD-Lo group for the following scales only: SCL anxiety, phobic anxiety, depression, somatisation and sleep problems.

The effect sizes for the YSQ domains were small to moderate (*d* = 0.39–0.54 and *d* = 0.36–0.58) and significant for the PD-Lo and PD-Hi groups respectively.

The effect sizes were small (*d* = 0.08–0.32 and *d* = 0.01–0.52) for the PD-Lo and PD-Hi groups respectively. The effects for the UCL scales *avoidance* and *palliative reaction pattern* were not significant in either patient group.

In conclusion, there were improvements in both patient groups at treatment termination and at follow-up in terms of psychiatric symptom severity, maladaptive schema severity and, to a lesser extent, coping styles. This was not the case for avoidance: the improvement in both patient groups was non-significant here and effect sizes were small.

Table 3 presents the results of the multilevel analysis to identify differences in outcome between the patient groups PD-Lo and PD-Hi. As can be seen, after Bonferroni correction, no differences in outcome were found at treatment termination.

**Table 3:** Differences in outcome between the PD-Lo and the PD-Hi patients at treatment termination (week 20) and three-month follow-up (week 32)

	week 20		week 32	
Outcome measure	***EMD (SE)***	***p***	***EMD (SE)***	***p***
SCL scales				
*anxiety*	−0.61 (0,93)	.51	−1.78 (1.02)	.08
*phobic anxiety*	−1.24 (0.54)	.02	− 1.21 (0.59)	.04
*depression*	−5.12 (1.98)	.01	−7.44 (2.09)	.00*
*somatisation*	−0.86 (0.93)	.36	−1.02 (1.03)	.32
*insufficiency*	0.44 (0.84)	.60	−0.66 (0.91)	.47
*interpersonal sensitivity*	−1.10 (1.45)	.45	−3.53 (1.57)	.03
*hostility*	0.61 (0.48)	.21	0.03 (0.53)	.96
*sleep problems*	0.16 (0.39)	.69	−0.38 (0.43)	.44
*other*	−0.67 (0.64)	.29	−2.76 (0.69)	.00*
*General Symptom Inventory*	−12.09 (6.64)	.07	−23.61 (7.17)	.00*
YSQ domains				
s*chema severity*	0.02 (0.08)	.81	0.03 (0.09)	.72
*disconnection-rejection*	0.05 (0.08)	.54	0.003 (0.10)	.98
*impaired autonomy*	−0.48 (0.08)	.56	−0.0002 (0.09)	.99
*impaired limits*	0.10 (0.07)	.19	0.12 (0.08)	.14
*other directedness*	0.12 (0.10)	.19	0.10 (0.10)	.34
*overvigilance and inhibition*	−0.01 (0.08)	.95	−0.04 (0.09)	.66
UCL scales				
*active coping*	0.49 (0.42)	.25	0.74 (0.47)	.11
*avoidance*	0.26 (0.47)	.59	0.57 (0.52)	.27
*palliative reaction pattern*	−0.26 (0.52)	.96	0.77 (0.58)	.18
*seeking social support*	0.25 (0.44)	.57	0.35 (0.48)	.47

*PD-Lo* Personality Disorder with low severity of depressive symptoms; *PD-Hi* Personality Disorder with high severity of depressive symptoms; *SCL* Symptom Checklist; *YSQ* Young Schema Questionnaire; *UCL* Utrecht Coping List; *EMD* Estimated Mean Difference; *SE* Standard Error. The minus sign(−) before the EMD score means that the PD-HI improves more than the PD-Lo patient group. *p < .01

However, at follow-up, a more favourable effect was reported for general symptoms (SCL-GSI) in the PD-Hi patient group. This was also the case for depression symptoms (SCL depression) and other unspecified symptoms (SCL *other problems*).

### Remission at treatment termination and three-month follow-up

At post-treatment, 50 % (76/152), of the total sample who completed therapy achieved reliable change as calculated using the Jacobsen and Truax method. Symptom remission based on the SCL-90 was achieved in 26.3% (40/152) of the patients. No statistical difference was found for reliable change between PD-Lo and PD-Hi patients: 44.9% (40/89) in PD-Lo patients and 57.1% (36/63) in PD-Hi patients. However, the remission rate was 32.6% (29/89) for the PD-Lo group and 17.5% (11/63) for the PD-Hi group, which is a significant difference (χ2(1) = 4.351, *p* = 0.04).

At follow-up, reliable change was observed in 44.6% (54/121) of all patients: 32.4% (23/71) in the PD-Lo group and 62.0% (31/50) in the PD-Hi group. The difference was significant (χ2(1) = 10.406, *p* = 0.001). Remission was achieved in 22.3% (27/121) of the patients: 21.1% (15/71) for the PD-Lo group and 24.0% (12/50) for the PD-Hi group. This was not a significant difference (χ2(1) = 0.140, *p* = 0.71).

## Discussion

The aim of this study was to see whether the presence of depression symptoms reduces responsiveness to SCBT-g in a broad sample of personality-disordered patients*.*

We found that this therapy was moderately effective in terms of bringing about improvements in psychiatric symptoms and maladaptive schemas. It proved to be more difficult to achieve improvements in coping styles, particularly in the avoidance style. There were hardly any differences in effect sizes between patients with or without comorbid depressive symptoms.

However, symptom remission was achieved in a minority of all patients, which may indicate this type of short-term group therapy could be seen as a valuable first step in a stepped-care model.

### Differences in baseline characteristics between patients with low or high severity of depressive symptoms

Personality disordered patients with severe depressive symptoms had more psychiatric problems, maladaptive schemas and coping, and more of them had received treatment previously. A vast majority of the PD-Lo patient group had also received treatment previously. Taken in conjunction, these data indicate that, in general, the patients in this study were rather difficult to treat, particularly those in the PD-Hi patient sample.

This baseline severity in more severely depressed patients was also reported by Renner et al. [14], who stated that patients with comorbid depression were more disturbed at the level of symptoms and personality pathology.

There were no differences between the two patient groups with regard to drop-out numbers and moment during treatment (i.e. early drop-out or late drop-outs). This is in line with the finding that there is still no homogeneous predictor for drop-out [31].

### Comparing treatment outcomes after short-term schema group therapy for personality disorders

Comparable studies (11. 7, 8, 32) mostly report a slightly higher effect on psychiatric symptom severity, with small [28] to high [7] effect sizes, than in our study.

This observation can be interpreted by reference to methodological differences. The naturalistic study of Jensen et al., [8], for example, applied a higher dose of the examined therapy (39 sessions) than our study (20 sessions).

Vreeswijk et al. [11] used a higher SCL-GSI cut-off score for remission on the basis of norm group data provided in the Dutch manual for SCL-90 of Arrindell & Ettema [20].

In addition, there are indications that symptoms in the samples of these studies were less severe than in our study. The patients in the study by Vreeswijk et al. [11] had a lower baseline symptom severity score (SCL-GSI = 188.87) and the study by Renner et al. [7] included patients with less severe symptoms (personality disorder or meeting subthreshold criteria for DSM-IV personality disorder) and the majority of the patients in the research sample of the Lorentzen study [32] did not have a personality disorder.

In general, improvements in coping styles seem to be more difficult to achieve, in particular for the coping style *avoidance*.

There are therefore strong indications that it is more difficult to address this area effectively with this short-term group approach. It is known that avoidant coping styles like self-distraction and disengagement aggravate personality disorders [33]. We therefore believe that treatment should focus more on avoidance in future approaches by including specific experiential or behavioural interventions such as role-playing or exposure to in vivo interventions.

### The impact of comorbid depressive symptoms on treatment outcome

Although the PD-Hi patient group had more severe baseline psychopathology, both patient groups achieved similar levels of reliable change. While remission was seen in significantly more PD-Lo patients at treatment termination, this difference in remission was no longer present at follow-up because more patients with comorbid depression improved during the follow-up period. This suggests that patients in the PD-Hi group, possibly as a result of a significant higher baseline psychopathology, need more time to recover than their counterparts in the PD-Lo group. This is in line with the study of Renner et al. [14], who came to the conclusion that it is not the comorbid depression but the high baseline psychiatric symptomatology in the comorbidity group that has a negative effect on treatment outcome.

In summary, despite the low symptom remission rates, schema therapy is a favourable treatment option for a broad group of patients, as suggested by other studies [14, 34]. The presence of depressive symptoms should not preclude referral to a group schema therapy programme with an exclusive focus on personality disorders. It is therefore reasonable to conclude that short-term therapy can be a beneficial first step, albeit one that will not ultimately be adequate for many patients.

### Limitations and strengths

Firstly, there was no control group in this study. We cannot therefore state the extent to which improvement after treatment was attributable to the schema group therapy or to natural symptom variations over time. However, it is important to bear in mind that the evaluated intervention was carried out in a complex patient population with long-standing problems who had almost all received apparently unsuccessful treatment in the past. We would therefore expect natural variation to result in only limited improvement in these patients. This was also suggested by a study of patients with a range of psychiatric disorders in Germany and Denmark: effect sizes ranging from 0.12 to 0.19 were reported when the patients were on a waiting list [35, 36].

The second shortcoming in our study is that the presence of a personality disorder was determined by a regular clinical intake procedure and not by structured diagnostic interviews such as the SCID-PD. On the other hand, all patients were referred specifically to our specialised service for the treatment of PD.

The severity of comorbid depressive symptoms was determined solely with a self-report questionnaire, which implies a slight risk of over-reporting [12]. In addition, the depressive symptom endorsement in self-report questionnaires from the PD population might reflect acute dysphoric distress rather than true depression.

Thirdly, we could not control for possible additional treatment during the follow-up period and so we cannot state to what extent this affected outcome at follow-up.

The main strength of this study was the strong ecological validity because of the naturalistic clinical setting. This indicates that the results could be generalised to regular clinical practice. Secondly, we had a large sample, resulting in high power and a smaller confidence interval and therefore in strong result validity and reliability. In addition, the large sample made it possible to perform the subgroup analysis of patients with more and less severe co-morbid depressive symptoms. As depressive symptoms are very common in patients with personality disorders, this also supports the validity of the results. Finally, treatment was delivered in groups which are cost-efficient and potentially applicable to multiple settings of clinical practice.

## Conclusions

The primary and secondary results indicate that a short-term form of schema therapy in groups can be an effective approach for a broad group of patients with personality disorders, including those with severe comorbid depressive symptoms, since it can lead to improvements not only in symptoms but also in underlying schemas.

Nevertheless, we should stress that the majority of patients did not achieve symptom remission. In particular, patients with more severely comorbid depressive symptoms may need higher doses or more intense treatment. We therefore believe that, for these complex patients, a short-term group approach is, above all, a helpful and pragmatic first step in a stepped-care model. In patients who have not achieved remission, more intensive or long-term forms of psychotherapy should be considered.

## Data Availability

The dataset and materials generated and analysed during the current study are not publicly available due to ethical restrictions and personal data protection. However, they are available upon the reasonable request to the corresponding author.

## Abbreviations

CSC: Clinical significant change
DSM-IV: Diagnostic and statiscal manual of mental disorders-IV
*d*: Effect size
PD: Personality disorders
PD-Lo: Personality disorder with low severity of depressive symptoms
PD-Hi: Personality Disorder with high severity of depressive symptoms
SCBT-g: Schema Cognitive behavioural therapy in groups
SCL-90-R: Symptom check List-90-Revised
SCL-90-GSI: Symptom checklist-90-general severity index
UCL: Utrecht coping list
YSQ: Young schema questionnaire
